# The nurse educator multiplier: Mobile-friendly, bite-sized training

**DOI:** 10.1097/nmg.0000000000000093

**Published:** 2024-02-05

**Authors:** Heather Theaux, Brian Johnson, Kashvi Patel, Ty Underwood

**Affiliations:** At NorthBay Medical Center in Fairfield, Calif., **Heather Theaux** is director of the Emergency Services/Trauma/Stroke/Behavioral Health Programs, and **Brian Johnson** is clinical manager of the Emergency Department/EDE Observation Unit. **Kashvi Patel** is an MD/MBA candidate at the University of Toledo College of Medicine and Life Sciences in Toledo, Ohio. **Ty Underwood**, formerly a lead ED technician at NorthBay Medical Center in Fairfield, Calif., is currently a client success manager for Elemeno Health in Oakland, Calif.

## Abstract

This learning solution shortens orientation times, reduces costs, and improves outcomes

## Abstract 2

A healthcare system implemented a mobile-friendly microlearning solution in their ED. This approach engaged the workforce, improved patient outcomes, shortened time to competency for new hires, and resulted in a cost savings of $849,000 per year.

**Figure:**
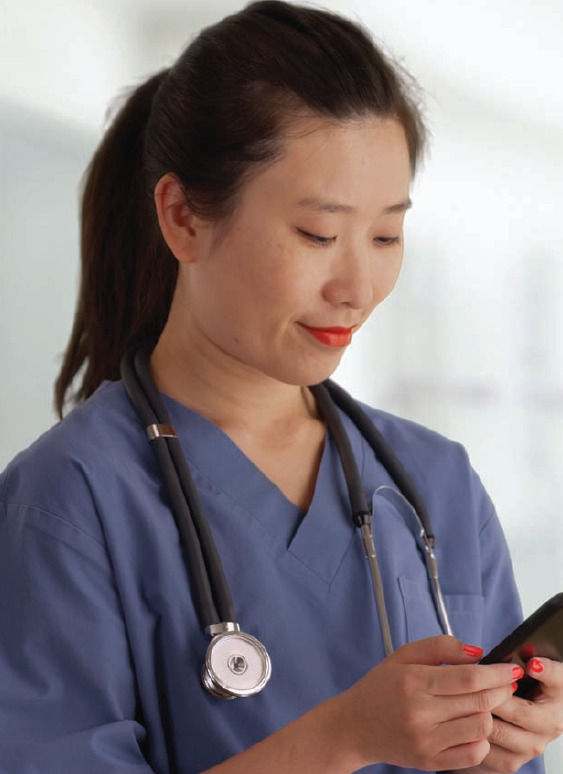
No caption available.

There's a dire need to streamline onboarding and orientation for both staff and management. The Tiered Skills Acquisition Model (TSAM) is a method of precepting with full patient loads and relies on observation by the orientee for learning; it's effective in shortening orientation for new graduates and experienced hires.^1^ This project looked at augmenting the TSAM with a just-in-time microlearning reference for those at the bedside delivering care. In essence, it took items that previously needed to be memorized, especially for low-frequency, high-risk tasks (such as Belmont infuser, arterial line setup, ST elevation myocardial infarction [STEMI] activation process), and transformed them into bite-sized support resources immediately accessible at the point of care.

## Background

Workforce shortages are the top concern for nurse leaders nationwide. The pandemic has multiplied the challenges frontline nurses face, combining growing stress and burnout with the Great Resignation, travelers, and inexperienced new graduates, resulting in both higher costs and increased patient harm.^2^ Managers are challenged: on one hand they must keep all of their staff up to date on constantly changing practices, workflows, and supplies; on the other hand, they must give extra attention to inexperienced new graduates with a pandemic-driven paucity of in-person training. And as managers are pulled into staffing, there's even less leadership to support an increasingly unstable workforce.

In one ED, a nurse leader addressed these challenges by deploying a mobile-friendly microlearning app to inform and upskill the ED workforce with customized, hyperlocalized, just-in-time support. Before implementation:

- The hospital IT department placed the website icon on every desktop in the ED.
- Staff completed an informal learning needs assessment that revealed various “pain points” at the bedside, which helped the nurse leader prioritize content.
- ED management together with the Clinical Education Department ranked needed information to be digitized into bite-sized resources that focused on pictorial information and checklists.

Implementation included the following:

- Unit leaders informed interdisciplinary staff about the app via email and department shift huddles, along with rounding and demonstrations.
- Introduction to the platform began with a “Wellness Challenge,” during which staff could attest to wellness activities and be entered into a drawing for a gift card.

By ensuring microlearning was both accessible and refreshable in the moment, this solution aimed to both train and sustain new learning.

## Methods

In early 2021, a cloud-based team microlearning app (Elemeno Health, Oakland, Calif.) was implemented in a 29-bed Level II trauma ED at a Magnet^®^-recognized hospital. End-users included all nurses, techs, and managers. This technology helped transform team communications and training into bite-sized, structured content that was deployed to frontline staff on both workstations and mobile devices. Workstation access was streamlined through a user email ID, password-free configuration; each personal mobile device retained the respective user ID to allow continual, single-click access. The vendor customized content to specific equipment, supplies, and workflows. The ED unit home page highlighted top resources recommended by management and allowed easy search by users, as well as personal bookmarking of favorites.

In 2019 through 2020, traditional orientation was reorganized in a TSAM approach to focus serially on skills, beginning with the most fundamental and, after demonstrating competency, progressing to steadily greater complexity while continuing to reinforce time management at every tier. Then, in 2021, the microlearning app was launched to deliver key, just-in-time job aids to supplement tiers within TSAM. Preceptors could now orient new staff in-context, sharing specific microlearning content with the orientee on mobile devices or workstations on wheels, while concurrently teaching a procedure or workflow in real time.

Orientees could subsequently access the same just-in-time training content repeatedly on-demand, reinforcing learning and supporting autonomy. Preceptors separately recorded (on paper) confirmation of orientee competency attainment at each tier before advancement to subsequent tiers. In-app orientee evaluations were separately developed by the vendor after the study period. Orientation concluded when orientees had acquired all tiered skills and demonstrated competency.

In this retrospective, Institutional Review Board-exempt improvement project, leaders in the ED compared orientation time (as reflected in timekeeping software) and costs for both nurses and techs for the intervals before (2019 to 2021) and after (2021 to 2022) implementation. They calculated the financial savings from hourly pay and benefits, along with the costs of backfilling 75% of otherwise open shifts with travelers, overtime, and on-call staff. The STEMI program nurse manager measured time-to-balloon for STEMI referrals during the same intervals, as this high-risk, complicated workflow involves both nurses and techs and reflects team performance quality.

## Results

**Figure 1:**
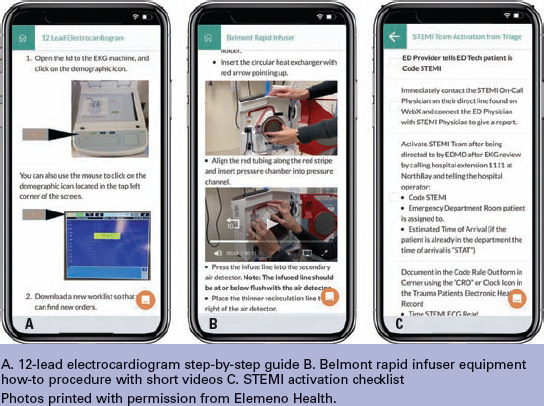
In-app microlearning examples

During the 16-month postimplementation period of the microlearning app, the team onboarded 95 new staff members and created 124 unique microlearning content items with over 1,000 views per month from both new and existing staff. Content included regular and emergent team updates (for example, the latest government guidance on COVID-19/monkeypox; hospital-specific news), high-risk, low-frequency practices (for example, rapid blood infuser), streamlined operational workflows (for example, STEMI receiving protocols) (see Figure 1).

**Figure 2:**
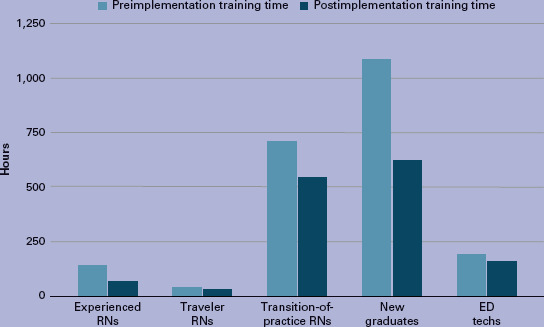
Comparison of average orientation time per staff type before and after implementation of the microlearning+TSAM orientation approach

Following implementation of the microlearning app and TSAM, 76 new nurses onboarded: 18 experienced ED RNs, 34 travelers, 15 transition-of-practice RNs (RNs moving from one specialty to a new one), and 9 new graduates. Orientation time to competency decreased 50% from 144 hours to 72 hours for experienced RNs (see Figure 2). This resulted in a savings of $6,200 per experienced RN (see Table 1). Orientation time decreased 29% (from 42 to 30 hours) for traveler RNs, with a savings of $2,300 per traveler. Orientation time decreased 24% (from 714 to 546 hours) for transition-of-practice RNs, with a savings of $14,600 per RN. Orientation time decreased 42% (from 1,092 to 630 hours) for new graduate RNs, translating to a savings of $40,000 per RN. Annualizing the total number of new RNs with salary and benefits, and including costs of backfill coverage ($244,000), the fiscal savings totaled $822,000 per year.

**Table 1:** Postimplementation orientation time and cost savings

Orientee	n	Average training time savings per orientee (hours)	% decrease vs. preimplementation baseline	Average training cost savings per orientee	Total training cost savings∗	Total backfill savings∗	Combined training and backfill savings∗	Annualized total orientation and backfill savings
Experienced RN	18	72	50	$6,200	$111,600	$39,700	$151,300	$124,000
Traveler RN	34	12	29	$2,300	$78,200	N/A	$78,200	$59,000
Transition-of-practice RN	15	168	24	$14,600	$219,000	$77,000	$296,000	$241,000
New graduate RN	9	462	42	$40,000	$360,000	$127,300	$487,300	$398,000
ED tech	19	36	18	$1,200	$22,800	$5,500	$28,300	$26,500
Grand total:							$1,041,100	$848,500

∗ During the 16-month study period

New tech orientations totaled 12 preimplementation and 19 postimplementation. Tech orientation time to competency decreased 18% from 196 to 160 hours. This translated to a savings of $1,200 per tech. Annualized savings, including reduced overtime and on-call coverage, totaled more than $25,000 per year. Combining the new hire ED RNs and technicians, this orientation approach saved 6,970 total hours per year or 3.4 full-time equivalents per year, which resulted in a combined yearly savings of nearly $850,000.

In both examples, it's worth noting that the preimplementation training time only involved the use of TSAM. The postimplementation training time represents the use of TSAM with the microlearning app.

Time-to-balloon for STEMI referrals was selected as an example of a high-risk, low-frequency event managed by the high-turnover techs. The vendor transformed the STEMI activation workflows into real-time guides delivered on the microlearning app. The STEMI referral microlearning guide was viewed 50 times by 31 unique users. Seventeen events occurred in the preimplementation interval and nine occurred postimplementation. Time-to-balloon decreased 20% from 123 minutes to 99 minutes. More generally, ED leadership reported greater compliance with the established workflows, which implies an increase in patient outcomes by shorter door-to-balloon times.

## Discussion

The nursing workforce is undergoing rapid change, not only in the mobility and turnover of staff, but also in their affinity for technology and the need to continually adapt to constantly evolving information specific to each department's respective environments. Based on their experiences in the consumer space, today's workers expect to find what they need, when they need it, in a multimedia format ready for consumption.^3^,^4^ By providing digital, bite-sized training at staff members' fingertips, nurse leaders can provide an agile platform for nursing education that can capture the teachings of institutional subject-matter experts and quickly adapt as hospital equipment, workflows, and practices continually evolve.

Nursing turnover is costly and carries an economic and noneconomic impact on staff and patients. Bae and colleagues estimated the direct dollar cost of turnover ranges from $21,514 to $88,000 per nurse in the US.^5^ Factors contributing to the economic cost of turnover include orientation and training, as well as loss of experienced staff and their collective organizational knowledge. This microlearning approach specifically addresses these gaps and can help decrease the financial burden from nursing turnover, with benefits of both reduced reliance on temporary labor and improving the ability to redeploy staff to other areas of need. Implementing TSAM in an adult inpatient medicine unit, Beamer and colleagues reduced budgeted new graduate RN orientation time by 38% and experienced RN orientation time by 25%, with cost savings of $5,300/RN and $2,050/RN, respectively.^6^

In the current study, complementing training with a microlearning approach augments both time and dollar savings, while engaging new staff with a communication and training solution, supporting them on a repeatable and sustainable basis throughout their staff tenure. Beyond economics, access to standardized, evidence-based practices is critical to error avoidance. The just-in-time contextual nature of microlearning drives rapid and widespread adoption of best practices, decreasing care variation and improving healthcare outcomes.

Additionally, easy access to information supports professional advancement. During the 16-month postimplementation period, 21 techs left the staff with 86% (18) advancing their healthcare careers by enrolling in nursing school (9), physician assistant school (3), or medical school (3). Techs reported that the transparency and contextuality of information available to all team members on the microlearning platform helped them to gain deeper familiarity with multidisciplinary practices.

## Implications for nurse leaders

This quality improvement project demonstrates successful deployment of an innovative and efficient solution to education and training for today's workforce. The significant fiscal savings from shortened orientation times, combined with the reduction of care variation, resulted in major resource-saving benefits for nurse leaders. This app-based microlearning approach can be leveraged as a “nurse educator extender” to help make up for diminishing resources.

As the app is cloud-based, sharing of specific microlearning content with other institutions is easy. Since implementing this solution for new hire orientation and general staff training, this ED has shared its format and specific content with other hospitals nationally, and in turn, has adapted and applied microlearning content from other institutions into its own.

Just as important as the orientation phase of competency is the ongoing competency and sustainability of the use of the app. With this in mind, the app is constantly updated with items staff identify as needed at the bedside. For instance, self-audits have been added to assist staff in low-frequency, high-alert documentation such as restraints and deep sedation. Management can monitor staff use of and engagement with the product. As such, future exploration should look at long-term retention and perhaps qualitative research of staff actual or perceived adoption and success.

## A shared success

With needed innovation in the onboarding process, layering a microlearning app in addition to the success of TSAM has optimized training time and, as a result, valuable training dollars. To better define the individual contribution of each, further study may be indicated.

This digital just-in-time microlearning approach has upskilled the frontline team, shortening the “door-to-floor” time for new hires, whether experienced staff or new grads; decreased costs; and improved patient outcomes. With broad staff engagement with and enthusiasm for this solution, and strong cost savings deployment is expanding to other units across the hospital system. As additional institutions share their own best practices, benefits can grow for all.
